# Chronic Myeloid Leukemia Presenting With Bilateral Optic Neuropathy and Sensorineural Hearing Loss as the First Clinical Presentation: A Case Report

**DOI:** 10.1155/crnm/9371576

**Published:** 2025-01-03

**Authors:** Sameen Ejaz, Rabia Nawaz, Fakeha Tariq, Ahmad Nawaz, Safia Bano, Ayesha Aslam, Fawad Khan, Zeeshan Ahmed, Aaqib Rashid, Sana Saqib

**Affiliations:** ^1^Department of Neurology, Mayo Hospital, King Edward Medical University, Lahore, Pakistan; ^2^Department of Pathology, Mayo Hospital, King Edward Medical University, Lahore, Pakistan

**Keywords:** adolescent, chronic myeloid leukemia, cranial neuropathy, optic neuropathy, sensorineural hearing loss, vision loss

## Abstract

Chronic myeloid leukemia (CML) is a myeloproliferative disorder that commonly manifests in chronic, accelerated, or blast phase. Typically observed in individuals aged 60–65 years, CML is infrequently diagnosed in adolescents. The usual presentation in late adulthood involves nonspecific symptoms such as fever, fatigue, and weight loss, with rare reports of initial neurological involvement. A 17-year-old male presented with bilateral vision loss and profound hearing loss, alongside a medical history marked by fever, night sweats, and weight loss. A positive tuberculosis contact raised suspicions of tuberculous meningitis, while cervical and inguinal lymphadenopathy suggested the possibility of neurosarcoidosis. Despite clinical signs pointing toward a neurological cause, elevated white blood cell (WBC) count, a bone marrow biopsy, and the identification of BCR-ABL translocation through chromosomal analysis surprisingly revealed a diagnosis of CML in the chronic phase. This case underscores the importance of considering hematological malignancy as a differential in cases of multiple cranial neuropathies, especially if supported by systemic symptoms. Understanding the diverse presentations of CML is essential for clinicians to provide timely and appropriate interventions particularly in young patients where it could mimic other neurological disorders leading to diagnostic challenges and delay in treatment initiation.

## 1. Introduction

Chronic myeloid leukemia, also known as CML, is usually diagnosed around 60–65 years and is rather a rare disease among children and adolescents. CML constitutes 2% of all leukemias in children younger than 15 years, with an annual incidence of one case per million, and 9% of leukemias in adolescents between the ages of 15 and 19 years, with an annual incidence of 2.2 cases per million [1]. An elevated white blood cell (WBC) count as revealed by a routine blood count and an enlarged spleen on a general physical examination usually hint toward the diagnosis of CML. Nonspecific symptoms such as fatigue and weight loss are usually present [2]. Sometimes leukemic infiltrations of the CNS can occur that will present itself in various forms such as facial paralysis [3], hearing loss, or visual loss, though these are encountered rarely. Leukemia patients with otologic manifestations are seen in 15%–40% of cases. Unilateral or bilateral sensorineural hearing loss (SNHL), tinnitus, and dizziness are among the common symptoms encountered [4]. Apart from otologic manifestation, another rare presentation can be symptoms involving the optic nerve. Though these are rare and usually discovered incidentally but can present as blurring of vision or total vision loss [5]. Thus, CML is not a common disorder in young adults, and cranial nerve involvement, as first clinical presentation, is rarely reported. Given the rarity of this presentation, this case report would enhance the limited body of the literature currently available on the subject.

## 2. Case Presentation

### 2.1. History

A 17-year-old Pakistani male presented with a one-month history of progressive, bilateral painless visual loss, which began gradually and worsened without any associated ocular symptoms. Three weeks later, he developed sudden, progressive bilateral hearing loss, accompanied by vertigo but without ear discharge, fullness, or tinnitus. The patient also reported intermittent low-grade fever, night sweats, lethargy, and undocumented weight loss over the past year, along with headaches and irritability. He had a strong history of tuberculosis contact, as his father died of pulmonary tuberculosis 2 years ago. The symptoms were not associated with cough, shortness of breath, nausea, vomiting, altered bowel habits, or any symptoms of focal deficits, fits, higher mental function impairment, delirium, memory impairment, bladder/bowel incontinence, or any head trauma. In addition, there was no history of bleeding disorders, substance abuse, or smoking, though he had a history of unprotected sexual contact.

### 2.2. Examination

The patient appeared lethargic, pale, and had a below-average build. He was vitally stable except for a temperature of 100°F. There was nontender bilateral cervical lymphadenopathy. Upon neurological examination, the motor system was intact. The sensory system and higher mental functions could not be assessed due to the patient's severe hearing and vision loss. Cranial nerve examination showed bilateral impairment of the optic and vestibulocochlear nerves. Ophthalmologic examination revealed dilated pupils with sluggish light reflexes, impaired color vision, and visual acuity limited to hand movements bilaterally. Fundoscopy revealed small deep retinal hemorrhages with blurred optic disc margins and obscuration of all vessels suggesting Grade V papilledema. The anterior eye segment was normal, with no evidence of vasculitis, Roth spots, or cotton wool spots. Pure tone audiometry indicated profound bilateral SNHL. The rest of the cranial nerves, cerebellar function, and systemic examinations were unremarkable. There were no signs of meningeal irritation, petechiae, bruises, or bone tenderness.

### 2.3. Investigations

**Figure 1:**
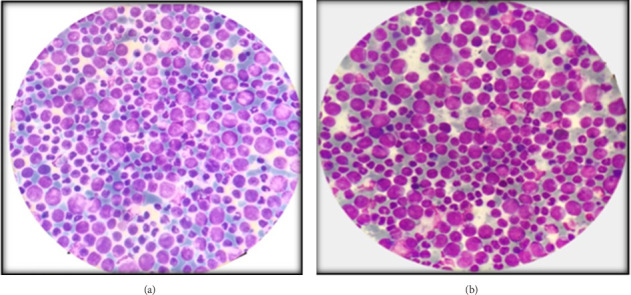
(a) Bone marrow aspirate showing hypercellularity and myeloproliferation with prominence of myelocytes and metamyelocytes. Myeloid: Erythroid ratio is increased. (b) Peripheral smear showing marked leukocytosis with left shift. A spectrum of myeloid cells is seen with a predominance of myelocytes.

**Figure 2:**
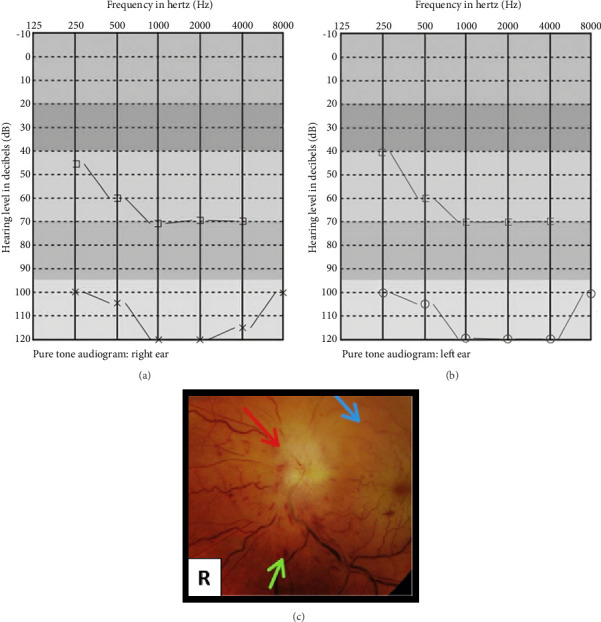
(a, b) Pure tone audiogram depicting profound sensorineural hearing loss in both ears. (c) Fundoscopy image of right eye showing deep retinal hemorrhages (green arrow), blurring of optic disc margins with obscuration of all vessels on disc suggestive of Grade V papilledema (red arrow) and pale retina as compared to other segments of retina secondary to vessel occlusion (blue arrow).

**Figure 3:**
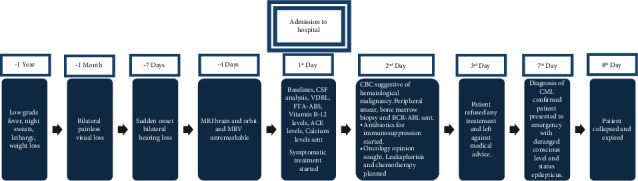
Timeline of events.

The complete blood count revealed a leukocyte count of 407 × 10^3^/*μ*L, along with bicytopenia (platelet count: 109 × 10^3^/*μ*L and red blood cells: 1.88 × 10^6^/*μ*L). The erythrocyte sedimentation rate was elevated at 45 mm/hour. A peripheral smear showed marked leukocytosis with immature granulocytes, indicating CML. PCR testing confirmed the presence of the BCR-ABL oncogene, and a bone marrow biopsy confirmed CML in the chronic phase, showing hypercellularity with prominent myelocytes and metamyelocytes. Ultrasound findings included mild hepatomegaly (16 cm), splenomegaly (13 cm), and bilateral cervical lymphadenopathy. Cerebrospinal fluid cytology revealed a few neoplastic cells with normal chemistry. Additional tests, including blood cultures, urine analysis, renal and liver function tests, serum electrolytes, coagulation profile, RPR/VDRL, serum FTA-ABS, vitamin B12 levels, T-spot TB test, calcium, and ACE levels, were all within normal limits. Imaging studies, including chest radiograph, ECG, EEG, MRI, and MR venography of the brain and orbit, were also unremarkable (Figures 1, 2, and 3).

### 2.4. Treatment

The initial management strategy revolved around symptomatic treatment, but as soon as the diagnosis of CML was established, the patient was referred to the oncology department for immediate leukapheresis and chemotherapy. The symptomatic treatment included acetaminophen 1 g intravenously twice a day for fever and night sweats. Empiric antibiotic therapy was also started for immunocompromised state.

### 2.5. Diagnostic Challenges

Initially, at the time of presentation, primary and secondary neurological causes were considered that included the following:

1. Tuberculosis meningitis: Presence of headache, undocumented chronic low-grade fever, night sweats, weight loss, lymphadenopathy, poor socioeconomic status, malnutrition, and positive tuberculosis contact along with multiple bilateral cranial neuropathies supported tuberculous meningitis as one differential, but unremarkable chest radiograph and cerebrospinal fluid analysis made tuberculous meningitis unlikely
2. Neurosarcoidosis: The young age, bilateral optic and vestibulocochlear involvement, fatigue, fever, night sweats, and cervical lymphadenopathy suggested neuroinflammatory disorder such as neurosarcoidosis but a normal chest radiograph, calcium, and angiotensin-converting enzyme levels ruled out neurosarcoidosis
3. Neurosyphilis: History of unprotected sexual contact, fever, night sweats, and sudden onset multiple cranial neuropathies was suggestive of neurosyphilis, but it was ruled out due to negative VDRL test and FTA-ABS

The complete blood count report proved to be a turning point and established the diagnosis of some hematological malignancy. The peripheral smear showed marked leukocytosis with immature granulocytes, eosinophilia, and basophilia, suggesting CML, which was confirmed by a positive BCR-ABL oncogene test. A subsequent bone marrow biopsy confirmed CML in the chronic phase. The diagnosis was particularly challenging due to the rare presentation of CML as multiple bilateral cranial neuropathies in a young adult.

### 2.6. Outcome

The patient and his family were counseled regarding the possibility of a hematological malignancy and were advised to have an oncological consultation, but he left the hospital in a denial phase. He later presented to the emergency department with status epilepticus and aspiration pneumonia after 4 days. He was managed accordingly but developed respiratory failure and was shifted to the mechanical ventilator. Unfortunately, he could not survive.

## 3. Discussion

CML is a type of myeloproliferative neoplasm involving uncontrolled proliferation of myeloid cells at different stages of maturation. CML may present in chronic phase, accelerated phase, blast phase, or blast crisis. Most of the patients present in the chronic phase of CML. Chronic phase of CML may be totally asymptomatic in half of the patients or may present with nonspecific symptoms such as fever, fatigue, and weight loss [6]. There is a wide diversity of presentation of CML but neurological involvement on initial presentation has been rarely reported [7]. Neurological involvement in CML is rare, but there is evidence of leptomeningitis that is the most common of all neurological presentations of CML. Other presentations include extra-axial hemorrhage or formation of mass in dura or parenchyma [8]. Eye symptoms are the initial presentation in only 5%–10% of patients diagnosed with CML, and patients with these symptoms are reported to have a lower 5-year survival rate than those without these manifestations [9]. This is also exemplified in this case, where the patient expired shortly after being diagnosed. In the given case, patient presented with bilateral cranial neuropathies that eventually turned out to be due to CML in the chronic phase, making it a very unusual presentation of CML.

The exact cause of the vision and hearing loss remains uncertain, but several mechanisms may be suggested to account for this uncommon presentation. One potential mechanism of bilateral vision loss could be leukemic cell infiltration of the optic nerves. Interestingly, a study conducted by Schocket et al. [10] has reported a case where leukemic infiltration of the optic nerve occurred despite a normal appearance of optic nerve on MRI. This suggests that such infiltration may not always be detectable through standard imaging techniques. Similar reasoning could be applied to explain the observed hearing loss, implicating leukemic infiltration of the vestibulocochlear nerve as a potential cause. Another possible mechanism could be the extremely high WBC count as there is a high possibility of hyperviscosity syndrome with WBC count more than 300 × 10^9^/L [11]. Such a significant increase in blood viscosity may have led to reduced blood flow and high risk of thrombosis, particularly affecting the microcirculation resulting in ischemia and neuropathy. Similar mechanisms have been suggested by Costagliola C. et al. with reference to optic nerve involvement in CML [12]. We recommend conducting a comprehensive ophthalmic and otologic examination for all CML patients, regardless of whether they exhibit symptoms. Similarly, every patient presenting with unexplained ophthalmic or otologic symptoms should undergo a complete blood count to rule out any hematological malignancy. This case also suggests that patients of CML presenting with such manifestations have a poor prognosis.

The uniqueness of this case report lies in the uncommon nature of its presentation. The patient exhibited cranial neuropathies as the initial symptoms at a young age, initially leading us to consider more common pathologies. However, through meticulous history-taking, thorough physical examination, and comprehensive diagnostic evaluation, we were able to pinpoint the rare diagnosis of leukemic optic and vestibulocochlear neuropathy stemming from CML. While this case report successfully identified the condition early on, its impact was hindered by the inability to proceed with the planned management and evaluate the patient's response to treatment. Although it adds valuable insight to the medical literature, the limitation of not being able to administer therapy and assess its response highlights the need for further exploration. Future research endeavors should aim to elucidate the definitive mechanism underlying optic and vestibulocochlear nerve involvement and explore optimal therapeutic interventions. By delving into these areas, we can broaden our understanding and pave the way for more effective treatment strategies in similar cases.

## 4. Conclusion

It is crucial to acknowledge the necessity of considering CML and other hematological malignancies as potential differentials when assessing a young patient with cranial neuropathies, rather than solely focusing on excluding primary neurological disorders. This emphasizes the importance of conducting a complete blood count to screen for such conditions. The key takeaway from this case report is that neurologists, general practitioners, and hematologists should all remain vigilant for the possibility of CML in young adults presenting with cranial neuropathies as their initial symptoms. Early recognition of this potential diagnosis can facilitate prompt treatment and ultimately improve the patient prognosis.

## Data Availability

The data supporting the findings of this case report can be provided upon reasonable request to the corresponding author. The data have been anonymized to ensure patient confidentiality and adhere to ethical standards.

## References

[1] Hijiya N., Schultz K. R., Metzler M., Millot F., Suttorp M. (2016). Pediatric Chronic Myeloid Leukemia Is a Unique Disease that Requires a Different Approach. *Blood*.

[2] Chronic Myelogenous Leukemia Clinical Presentation: History, Physical Examination [Internet]. *Emedicine.medscape.com*.

[3] Khajeh A., Miri Aliabad G., Fayyazi A., Soleimani G., Keikha R. (2018). Chronic Myelogenous Leukemia Presenting with Facial Nerve Palsy in an Infant. *Iranian Journal of Child Neurology*.

[4] Syamsuddin I. K., Notopuro P. B. (2022). Sensorineural Hearing Loss in Juvenile CML: A Rare Case Report in Surabaya, Indonesia. *International Medical Case Reports Journal*.

[5] Nagpal A., Moharana B., Sharma R., Sharma B. (2022). Ischemic Optic Neuropathy in Chronic Myelogenous Leukemia Presenting as Unilateral Optic Disc Edema. *Odisha Journal of Ophthalmology*.

[6] Minciacchi V. R., Kumar R., Krause D. S. (2021). Chronic Myeloid Leukemia: a Model Disease of the Past, Present and Future. *Cells*.

[7] Berg S., Nand S. (2017). Neurological Complications of the Leukemias across the Ages. *Current Neurology and Neuroscience Reports*.

[8] Palejwala A. H., O’Connor K. P., Shi H., Villeneuve L., Scordino T., Glenn C. A. (2019). Chronic Myeloid Leukemia Manifested as Myeloid Sarcoma: Review of Literature and Case Report. *Journal of Clinical Neuroscience*.

[9] Al-Amri A., Buzaid A. (2017). Sudden Visual Loss as an Initial Manifestation of Chronic Myeloid Leukemia. *Saudi Journal of Medicine and Medical Sciences*.

[10] Schocket L. S., Massaro-Giordano M., Volpe N. J., Galetta S. L. (2003). Bilateral Optic Nerve Infiltration in Central Nervous System Leukemia. *American Journal of Ophthalmology*.

[11] Yassin M. A., Ata F., Mohamed S. F. (2022). Ophthalmologic Manifestations as the Initial Presentation of Chronic Myeloid Leukemia: a Review. *Survey of Ophthalmology*.

[12] Costagliola C., Rinaldi M., Cotticelli L., Sbordone S., Nastri G. (1992). Isolated Optic Nerve Involvement in Chronic Myeloid Leukemia. *Leukemia Research*.

